# Current status of an implant-based augmentation mammaplasty in Korean women: Implications for risk management and social sustainability of breast implant industry in the context of the first Korean case of a medical device fraud

**DOI:** 10.1097/MD.0000000000044092

**Published:** 2025-09-05

**Authors:** Robert Kim

**Affiliations:** a Department of Medical and Pharmaceutical Affairs, Doctor CONSULT, Seoul, Korea.

**Keywords:** anaplastic, breast implants, fraud, large-cell, lymphoma, medical device recalls, safety-based medical device withdrawals

## Abstract

Stakeholders in the breast implant industry in Korea have recently experienced a crisis from breast implant-associated anaplastic large cell lymphoma and the first Korean case of a medical device fraud. We compared the short-term safety between the microtextured devices that are commercially available after the occurrence of breast implant crisis in Korea. The current study was conducted in a cohort of Korean women who had received an implant-based augmentation mammaplasty for aesthetic purposes between November 14, 2020 and October 13, 2022. We considered risk factors of complications in analyzing the safety of devices for the current study. A total of 801 Korean women (n = 801) were finally assessed. Incidences of capsular contracture were 1.79% (3/168), 3.64% (21/577), 8.11% (3/37), and 10.53% (2/19) in the patients receiving Motiva Ergonomix, Sebbin Sublimity, Sebbin Integrity, and Eurosilicone Round Collection, respectively. These differences reached statistical significance (*P* < .05). There were 2 women with rupture after receiving Sebbin Sublimity, although there were no cases of rupture in association with other brands of breast implants. Overall capsular contracture-free survival was estimated at 681.470 ± 8.314 (95% confidence interval [CI] 665.174–697.766) days. By breast implants, it was 708.899 ± 8.595 (95% CI 692.053–725.745), 599.327 ± 6.607 (95% CI 586.378–612.277), 584.941 ± 22.965 (95% CI 539.931–629.952), and 572.492 ± 37.374 (95% CI 499.240–645.745) days in the patients receiving Motiva Ergonomix, Sebbin Sublimity, Sebbin Integrity, and Eurosilicone Round Collection, respectively, in the increasing order. In conclusion, our results indicate that Motiva Ergonomix Round SilkSurface is currently a relatively safer device as compared with others in the context of the first Korean case of a medical device fraud. The breast implant industry in Korea should be aware of the importance of the social sustainability in manufacturing a device.

## 1. Introduction

A medical device is defined as instrument, apparatus, implement, machine, appliance, implant, reagent for in vitro use, software, material or other similar or related article that are manufactured for diagnostic or therapeutic purposes.^[1]^ The medical device industry plays a pivotal role in improving both the health and quality of life of patients.^[2]^ It is therefore mandatory to ensure the safety and efficacy of the medical device used by both healthcare professionals and patients. Over past decades, the medical device has undergone a stringent quality assessment from a reactive practice to a complete quality assurance approach throughout its lifecycle.^[3]^

A breast implant is a type IV implantable medical device that is used in augmentation mammaplasty, and its shape and safety are of primary concern for both surgeons and patients.^[4]^ It has undergone technological innovation and design improvement over time. From the first to the fifth generation of a breast implant, each generation is characterized by the technological development of its components.^[5,6]^ Most recently, the external surface of the fifth-generation of a breast implant has a contact with viable tissue, and it is characterized by an irregular texture that is expected to stimulate tissue adherence to the implant and subsequently to avoid an anatomical misplacement of a device.^[7]^ Thus, a macrotextured breast implant with varying pore size and depth has been commercially available in the market.^[8]^ During the accumulation of more clinical evidences demonstrating the effects of surface texturization in reducing the occurrence of capsular contracture (CC), a macrotextured breast implant has been reported to have a causal relationship with the onset of breast implant-associated anaplastic large cell lymphoma (BIA-ALCL). Therefore, it has been imperative that a breast implant with a novel surface topography emerge. Manufacturers have commercialized a silicone gel-filled breast implant (SGBI) with a microtextured surface as an innovative technology that may lower risks of both CC and BIA-ALCL.^[9]^ But this has also been contradicted by other studies.^[10–14]^

Although a microtextured breast implant is currently considered a smooth device, it is differentiated from a traditional smooth one, as summarized in Table 1.^[15–18]^ Motiva Ergonomix (Establishment Labs Holdings Inc., Alajuela, Costa Rica) and BellaGel SmoothFine (HansBiomed Co. Ltd., Seoul, Korea) have been known as 2 representative brands of a microtextured device in Korea.^[16,19]^ The short-term safety of traditional smooth SGBIs and microtextured devices was evaluated by a recent study.^[18]^ In Korea, there were a crisis from BIA-ALCL and the first Korean case of a medical device fraud.^[16–18,20–22]^

**Table 1:** Summary of nanotextured or microtextured breast implants in Korea.

Manufacturer	Trade name	Year of release
Establishment Labs Holdings Inc. (Alajuela, Costa Rica)	Motiva Ergonomix	2016
HansBiomed Co. Ltd. (Seoul, Korea)	BellaGel SmoothFine	2017
GC Aesthetics PLC (Apt Cedex, France)	Eurosilicone Round Collection	2016
Groupe Sebbin SAS (Boissy-l’Aillerie, France)	Sebbin Integrity	2018
	Sebbin Sublimity (formerly the Naturgel)	2012

Despite great advancements in breast implant technology and surgical technique, a risk of postoperative complications poses a challenge for stakeholders in the breast implant industry (BII).^[20,23]^ Of such complications, both CC and rupture are observed the most prevalently. Still, however, little is known about the exact pathogenesis and pathophysiology of CC, although it may occur through a multifactorial process.^[24,25]^ Rupture is another concern that has a close relationship with the age of a breast implant. A continuous monitoring of patients receiving a device would therefore be mandatory to assess its long-term safety.^[26]^

Given the above background, we compared incidences of CC and rupture between the microtextured SGBIs after the occurrence of breast implant crisis (BIC) in Korea. Then, we discuss implications of the current results for risk management and sustainable development of BII in Korea in the context of the first Korean case of a medical device fraud.

## 2. Materials and methods

### 2.1. Literature review

#### 2.1.1. BIC in Korea

Recently, the BIC was a big issue to those who are involved in the BII in Korea; it can be classified as the first and second BIC.^[16–18,20–22]^

##### 2.1.1.1. The first BIC in Korea: BioCell textured device (Allergan Inc., Irvine)

There were 3 Korean cases of BIA-ALCL between 2019 and 2020 in relation to BioCell textured device (Allergan Inc., Irvine). Since August 29, 2019, no textured devices have been used, as mandated by the Korean Ministry of Food and Drug Safety (KMFDS).^[17,23]^

##### 2.1.1.2. The second BIC in Korea: BellaGel/BellaGel SmoothFine

**Figure 1.**
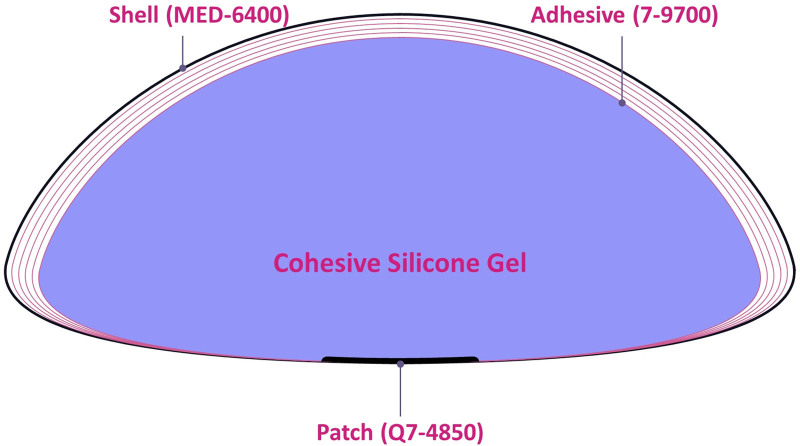
Use of unapproved materials for manufacturing of the BellaGel. A total of 5 constituents were not approved for use for manufacturing of the BellaGel implants; these include 7-9700 (soft skin adhesive), Q7-4850 (liquid silicone rubber), MED2-6300 (silicone gel), MED-6400 (silicone dispersion), and MED2-4213 (skin adhesive).

HansBiomed Co. Ltd. violated the regulatory requirement enforced by the KMFDS. The manufacturer used unapproved, harmful materials, such as 7-9700, Q7-4850, MED2-6300, MED-6400, and MED2-4213 (Fig. 1).^[27–30]^

HansBiomed Co. Ltd. deliberately modified the shell layer of BellaGel SmoothFine in violation of the regulatory requirement. The KMFDS approved the commercialization of BellaGel SmoothFine with a 5-layered shell, although the manufacturer illegally manufactured a 4-layered device to increase its soft feel.^[31,32]^

#### 2.1.2. The global impact of the first Korean case of a medical device fraud

##### 2.1.2.1. Europe

**Figure 2.**
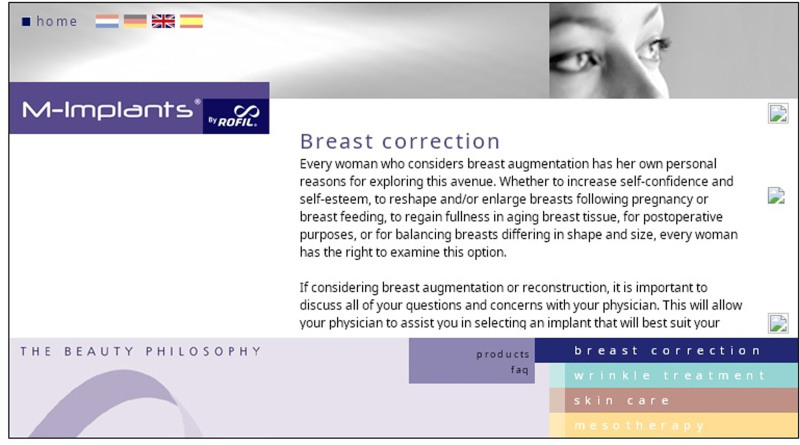
A snapshot of the website of the Rofil Medical Implants Ltd.

**Figure 3.**
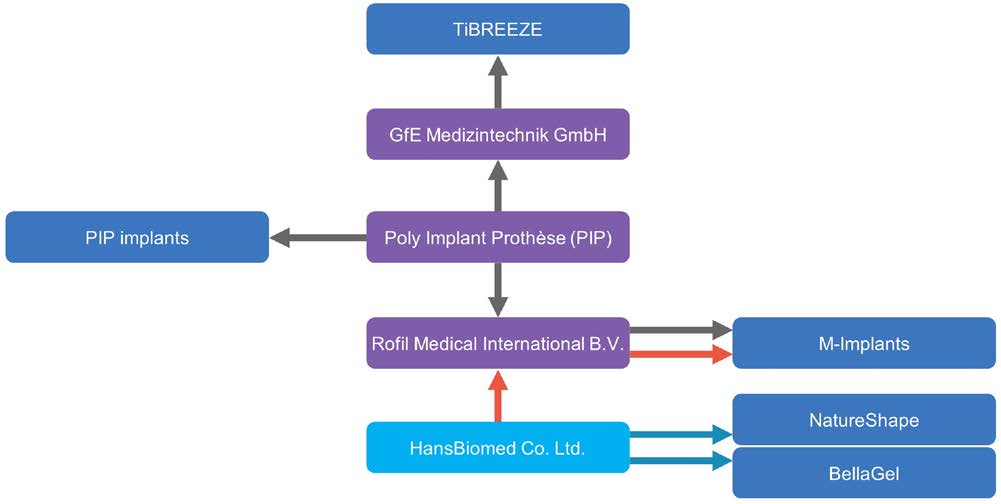
Involvement of the HansBiomed Co. Ltd. in the Poly Implant Prothèse fraud.

**Figure 4.**
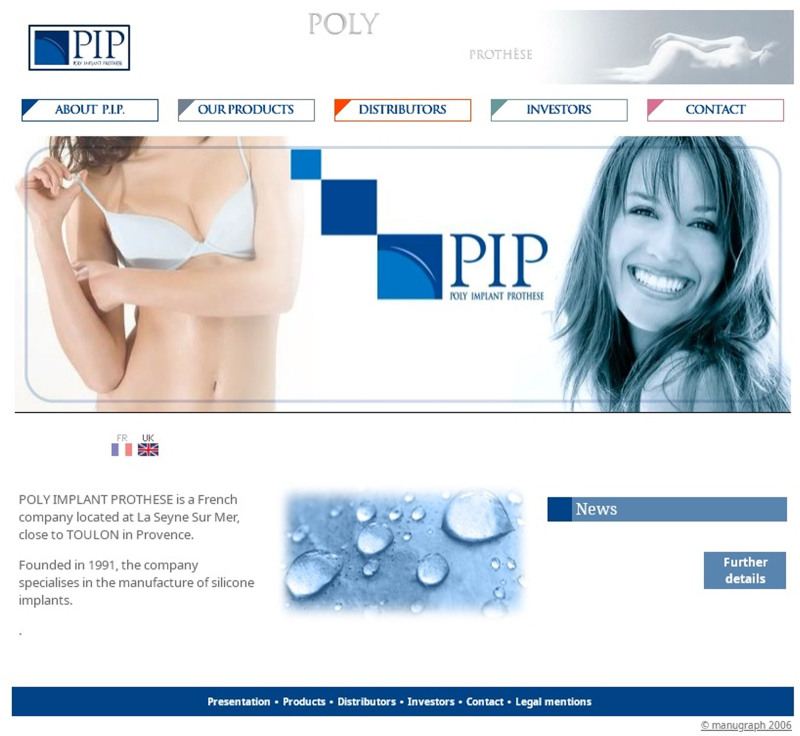
A snapshot of the website of the Poly Implant Prothèse.

In France, between 2010 and 2012, HansBiomed Co. Ltd. was involved in the Poly Implant Prothèse (PIP) fraud (Figs. 2 and 3).^[33–35]^ PIP was allegedly known as the third-largest global manufacturer of an SGBI from France (Fig. 4).^[36–38]^ Therefore, it deserves serious consideration that HansBiomed Co. Ltd. was involved in manufacturing the PIP breast implants under different brand names, such as M-implants.^[39–42]^

##### 2.1.2.2. China

According to the news report dated November 12, 2020, HansBiomed Co. Ltd. avoided being inspected by the National Medical Products Administration (NMPA, formerly China Food and Drug Administration) when entering the Chinese market for the commercialization of the BellaGel breast implants in China. After obtaining the regulatory approval for them from the KMFDS in the late 2015, HansBiomed Co. Ltd. accelerated the entry into the overseas market. The manufacturer finally obtained the regulatory approval of BellaGel Smooth from the NMPA in 2016.^[43]^

In October of 2018, HansBiomed Co. Ltd. held a press conference celebrating a launch of the BellaGel SmoothFine (formerly BellaGel Micro, BellaGel breast implant with a microtextured surface) in Seoul; the manufacturer had a plan to enter the Chinese market since 2019. Nevertheless, the manufacturer had internal problems; the NMPA performed a rapid inspection of BellaGel breast implants that were commercially available in China in about June of 2018. In November of 2018, the manufacturer received the results from a certification testing institute located in China; a responsible officer of HansBiomed China Inc. (Shanghai, China) urgently sent an e-mail to the head office in Seoul, Korea, who reported that BellaGel breast implants did not pass the test for evaporation residue, trace element, and shell thickness. There were considerable differences in criteria for accepting the quality of breast implants based on impurities on the evaporation residue test between China and Korea. In more detail, only the sample is extracted from the device and then assayed for impurities in Korea. In China, however, the sample is boiled and the residue from the vapor is assayed accordingly. Based on the results showing that BellaGel breast implants did not pass trace element test, it can be inferred that they contain heavy metals at higher levels than the standard limit. But the manufacturer did not identify any problems from raw materials. The manufacturer therefore concluded that it would be impossible to pass the test even if a retest is performed.^[43]^

Nevertheless, the manufacturer decided to resolve the issue using “*Guanxi*,” referred to as a direct financial relationship with the Chinese government, by asking officials of the NMPA to falsify the results of the test and estimated the payment at approximately CNY 15,000,000.^[43]^

#### 2.1.3. Changes in the Korean breast implant market after the occurrence of BIC in Korea

**Figure 5.**
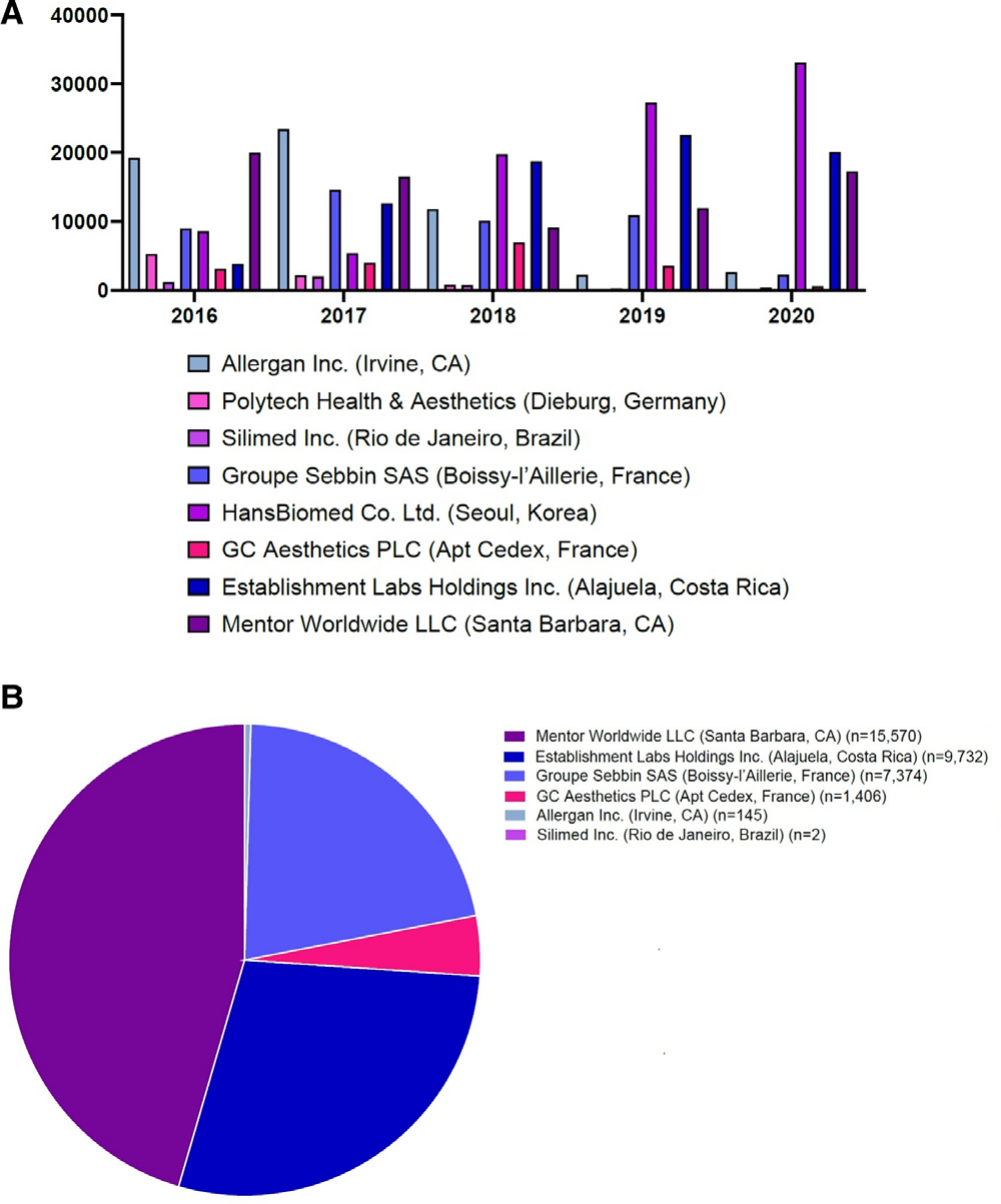
The annual number of breast implants that were circulated in the Korean market over a 6-year period (2016–June 2021). (A) The amount of sales between 2016 and 2020 and (B) the market share of a device between January and June, 2021.

According to the KMFDS statistics, approximately 77,000 breast implants are annually circulated in the Korean market between 2016 and 2020. After the occurrence of BIC in Korea, however, there were notable changes in the market; Mentor Worldwide LLC. was the manufacturer whose products were sold the most prevalently (n = 15,570). This is followed by Establishment Labs Holdings Inc. (n = 9732), Groupe Sebbin SAS (n = 7374), GC Aesthetics PLC (n = 1406), Allergan Inc. (n = 145), and Silimed Inc. (n = 2) in the decreasing order (Table 2 and Fig. 5).^[44]^

**Table 2:** The annual number of breast implants that were circulated in the Korean market over a 6-year period (2016–June 2021).

Manufacturer	2016	2017	2018	2019	2020	June 2021
Allergan Inc. (Irvine, CA)	19,231	23,395	11,782	2251	2641	145
Polytech Health & Aesthetics (Dieburg, Germany)	5261	2169	804	12	No longer available	
Silimed Inc. (Rio de Janeiro, Brazil)	1187	2000	744	275	377	2
Groupe Sebbin SAS (Boissy-l’Aillerie, France)	8964	14,585	10,063	10,911	2276	7374
HansBiomed Co. Ltd. (Seoul, Korea)	8573	5396	19,752	27,252	33,067	No longer available
GC Aesthetics PLC (Apt Cedex, France)	3139	3991	6955	3536	586	1406
Establishment Labs Holdings Inc. (Alajuela, Costa Rica)	3848	12,635	18,717	22,565	20,078	9732
Mentor Worldwide LLC (Santa Barbara, CA)	19,994	16,509	9118	11,914	17,237	15,570
Total	**70,197**	**80,680**	**77,935**	**78,716**	**76,262**	**34,229**

In November 13, 2020, the KMFDS mandated a 6-month suspension of the manufacture and sales of BellaGel breast implants.^[45]^ According to the new report dated September 22, 2023, however, HansBiomed Co. Ltd. canceled a lawsuit against the KMFDS; the manufacturer announced that it would resume the BellaGel business in March of 2023.^[46]^

Patients receiving the BellaGel SmoothFine complained of the manufacturer’s fraudulent practices. But both the manufacturer and some plastic surgeons denied any causal relationships between the defective BellaGel SmoothFine and patients’ complaints.^[47]^ Many patients even filed a lawsuit against the HansBiomed Co. Ltd. and thereby sought monetary compensation for damages caused by the manufacturer. The amount of such compensation was known to reach approximately USD 3.7 million.^[47]^ Nevertheless, HansBiomed Co. Ltd. claimed that a finished product of BellaGel SmoothFine is safe because it does not emit toxic substances although the manufacturer admitted the illegal use of unapproved materials.^[47]^ But the manufacturer did not admit that it deliberately modified the shell structure during manufacturing process.^[48]^

### 2.2. Study patients and setting

The current multicenter, retrospective observational study was conducted in a cohort of Korean women who had received an implant-based augmentation mammaplasty for aesthetic purposes during a 2-year period between November 14, 2020 and October 13, 2022. Inclusion/exclusion criteria for the current study are summarized in Table 3.

**Table 3:** Eligibility criteria.

Eligibility criteria
*Inclusion criteria* (1) Women aged 22 years or older (2) Women who underwent primary augmentation mammaplasty using nanotextured or microtextured SGBIs for aesthetic purposes (3) Women with an adequate amount of tissue for coverage of the breast implant (4) Women with availability of follow-up data
*Exclusion criteria* (1) Women with unilateral or bilateral presence of premalignant breast lesions (2) Women with untreated malignancies (3) Women with an inadequate amount of or inappropriate tissue for coverage of the breast implant because of radiation-induced damage, vascular compromise or impaired wound healing (4) Women with abscess or infection (5) Women who had a past history of taking any drugs that may interfere with blood clotting or raise risks of developing postoperative complications (6) Women with underlying medical conditions that may raise risks of developing postoperative complications (*e.g.*, obesity [BMI ≥ 40], DM, autoimmune disease, chronic lung, severe cardiovascular disease, connective tissue or rheumatoid disease) (7) Women who are pregnant or breastfeeding (8) Women with medical conditions that may interfere with wound healing (*e.g.*, active infectious collagen disease) (9) Women with severe lung disease (*e.g.*, chronic obstructive pulmonary disease) (10) Women with active cutaneous or systemic infections (11) Women undergoing radiotherapy or chemotherapy within 6 months preoperatively (12) Women lost to follow-up

BMI = body mass index, DM = diabetes mellitus, SGBI = silicone gel-filled breast implant.

### 2.3. Ethics statement

We obtained the ethical approval of the current study from the Internal Institutional Review Board of the Korea National Institute of Bioethics Policy (Institutional Review Board approval #: P01-202101-21-021) and conducted it in compliance with the relevant guidelines and applicable laws. However, we failed to receive a written informed consent from the patients because of retrospective nature of the current study.

### 2.4. Treatment, follow-up and assessment of the patients

The patients were surgically treated and then regularly followed up, as previously described.^[17–19,49–51]^

We analyzed age, body mass index (BMI), follow-up (FU) period and incidences of the postoperative complications, such as CC and rupture.^[51,52]^

We excluded risk factors of postoperative complications, such as an FU period of <1 year, a BMI ≥ 30 kg/m^2^ and trans-axillary or peri-areolar incisions, in analyzing the safety of microtextured SGBIs.^[53–61]^

Data was presented as the number of the patients with percentage, mean ± standard deviation or mean ± standard error and then analyzed using the SPSS ver. 18.0 for windows (SPSS Inc., Chicago). Differences in incidences of postoperative complications between the microtextured SGBIs were analyzed using the repeated measures analysis of variance and Duncan post hoc analysis. Moreover, time to CC, CC-free survival and cumulative hazards were estimated. Log-rank test was also performed to assess the statistical significance and 95% confidence intervals (CIs) were provided. Statistical significance was set at *P* < .05.

## 3. Results

### 3.1. Baseline characteristics of the patients

A total of 801 Korean women (n = 801) were finally included in the current study; they received an implant-based augmentation mammaplasty using Sebbin Sublimity (n = 577), Motiva Ergonomix (n = 168), Sebbin Integrity (n = 37), and Eurosilicone Round Collection (n = 19). There were no significant differences in age, BMI and the length of FU period (*P* > .05) (Table 4).

**Table 4:** Baseline characteristics of the patients (n = 801).

	Values				*P*-value
	Sebbin sublimity (n = 577)	Motiva ergonomix (n = 168)	Sebbin integrity (n = 37)	Eurosilicone round collection (n = 19)	
Age (yr old)	32.14 ± 4.18	31.27 ± 5.47	28.36 ± 4.18	32.77 ± 3.89	>.05
BMI (kg/m^2^)	24.82 ± 0.04	24.79 ± 1.03	24.88 ± 1.27	25.01 ± 2.01	
FU (mo)	12.13 ± 0.11	12.77 ± 0.68	12.25 ± 0.18	12.64 ± 0.52	

Values are mean ± standard deviation.

BMI = body mass index, FU = follow-up.

### 3.2. Differences in incidences of postoperative complications between the breast implants

Incidences of CC were 1.79% (3/168), 3.64% (21/577), 8.11% (3/37), and 10.53% (2/19) in the patients receiving Motiva Ergonomix, Sebbin Sublimity, Sebbin Integrity, and Eurosilicone Round Collection, respectively, in the increasing order. These differences reached statistical significance (*P* < .05). There were 2 women with rupture after receiving Sebbin Sublimity, although there were no cases of rupture in association with other brands of breast implants (Tables 5 and 6).

**Table 5:** Incidences of postoperative complications (n = 801).

	Values				*P*-value
	Sebbin Sublimity (n = 577)	Motiva Ergonomix (n = 168)	Sebbin Integrity (n = 37)	Eurosilicone Round Collection (n = 19)	
CC	21 (3.64%)	3 (1.79%)	3 (8.11%)	2 (10.53%)	<.05*
Rupture	2 (0.35%)	0 (0.00%)	0 (0.00%)	0 (0.00%)	

Values are the number of the patients with percentage.

CC = capsular contracture.

* Statistical significance at *P* < .05.

**Table 6:** Differences in incidences of capsular contracture (n = 801).

Breast implants	N	n	Censored values
Sebbin Sublimity	577	21	556 (96.4%)
Motiva Ergonomix	168	3	165 (98.2%)
Sebbin Integrity	37	3	34 (91.9%)
Eurosilicone Round Collection	19	2	17 (89.5%)

n = incidences of capsular contracture, N = total number of the patients.

Results of log-rank test showed that incidences of CC were the significantly lowest in the patients receiving Motiva Ergonomix (*P* < .05) (Table 7).

**Table 7:** Results of log-rank test (n = 801).

Comparisons	*χ* ^2^	*P*-value
Sebbin Sublimity vs Motiva Ergonomix	4.600	.032*
Sebbin Integrity vs Motiva Ergonomix	5.466	.019*
Eurosilicone Round Collection vs Motiva Ergonomix	4.665	.031*

* Statistical significance at *P* < .05.

Overall, CC-free survival was estimated at 681.470 ± 8.314 (95% CI 665.174–697.766) days. By breast implants, it was 708.899 ± 8.595 (95% CI 692.053–725.745), 599.327 ± 6.607 (95% CI 586.378–612.277), 584.941 ± 22.965 (95% CI 539.931–629.952) and 572.492 ± 37.374 (95% CI 499.240–645.745) days in the patients receiving Motiva Ergonomix, Sebbin Sublimity, Sebbin Integrity, and Eurosilicone Round Collection, respectively, in the increasing order. This showed that Motiva Ergonomix achieved the longest survival in our series (Table 8).

**Table 8:** Capsular contracture-free survival (n = 801).

Breast implants	CC-free survival
Total	681.470 ± 8.314 (665.174–697.766)
Sebbin Sublimity	599.327 ± 6.607 (586.378–612.277)
Motiva Ergonomix	708.899 ± 8.595 (692.053–725.745)
Sebbin Integrity	584.941 ± 22.965 (539.931–629.952)
Eurosilicone Round Collection	572.492 ± 37.374 (499.240–645.745)

Values are mean ± standard error with 95% confidence intervals.

CC = capsular contracture.

**Figure 6.**
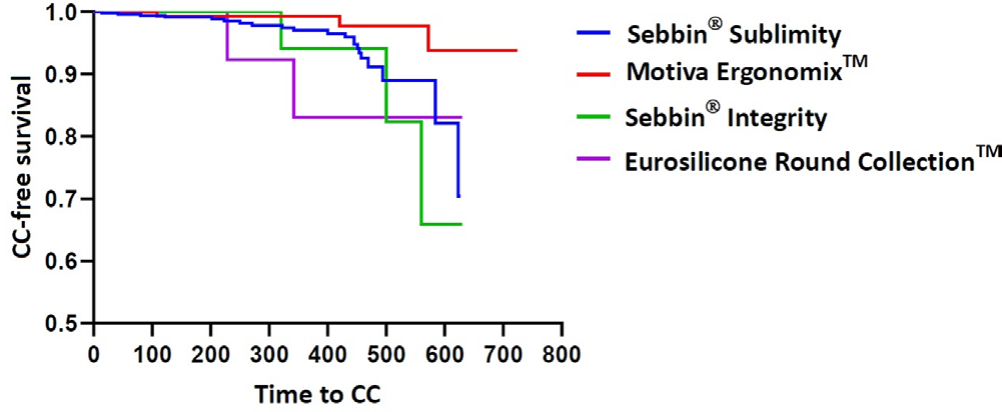
Kaplan–Meier survival (n = 801). CC = capsular contracture.

**Figure 7.**
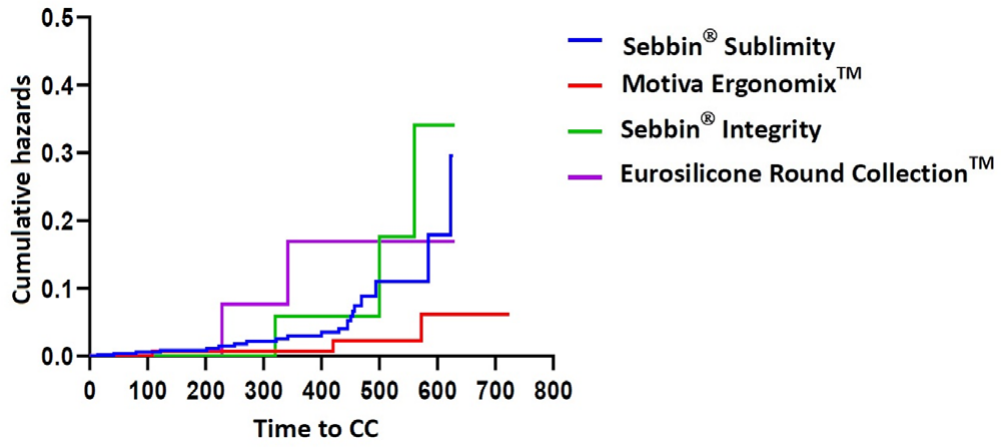
Kaplan–Meier hazards (n = 801). CC = capsular contracture.

Cumulative rates of CC are summarized in Table 9. Kaplan–Meier cumulative survival and hazards are plotted in Figures 6 and 7, respectively.

**Table 9:** Cumulative rates of capsular contracture (n = 801).

Breast implants	FU	N	n	Cumulative rates of CC
Sebbin Sublimity	14	526	1	0.998 ± 0.002 (0.994–1.000)
	42	490	1	0.996 ± 0.003 (0.991–1.000)
	80	452	1	0.994 ± 0.004 (0.987–1.000)
	122	410	1	0.991 ± 0.004 (0.983–1.000)
	202	324	1	0.988 ± 0.005 (0.978–0.999)
	223	310	1	0.985 ± 0.006 (0.973–0.997)
	250	292	1	0.982 ± 0.007 (0.968–0.996)
	271	278	1	0.978 ± 0.008 (0.963–0.994)
	322	244	1	0.974 ± 0.009 (0.957–0.992)
	342	230	1	0.970 ± 0.010 (0.951–0.989)
	400	190	1	0.965 ± 0.011 (0.944–0.986)
	430	177	1	0.959 ± 0.012 (0.936–0.984)
	445	162	2	0.948 ± 0.015 (0.920–0.977)
	451	142	1	0.941 ± 0.016 (0.910–0.973)
	454	131	1	0.934 ± 0.017 (0.900–0.968)
	457	117	1	0.926 ± 0.019 (0.889–0.964)
	469	64	1	0.911 ± 0.024 (0.866–0.959)
	494	42	1	0.890 ± 0.031 (0.830–0.953)
	584	13	1	0.821 ± 0.072 (0.692–0.975)
	623	7	1	0.704 ± 0.125 (0.497–0.997)
Motiva Ergonomix	108	141	1	0.993 ± 0.007 (0.979–1.000)
	420	63	1	0.977 ± 0.017 (0.944–1.000)
	572	25	1	0.938 ± 0.042 (0.860–1.000)
Sebbin Integrity	320	17	1	0.941 ± 0.057 (0.836–1.000)
	500	8	1	0.824 ± 0.121 (0.618–1.000)
	560	5	1	0.659 ± 0.176 (0.390–1.000)
Eurosilicone Round Collection	228	13	1	0.923 ± 0.074 (0.789–1.000)
	342	10	1	0.831 ± 0.110 (0.641–1.000)

Values are mean ± standard error with 95% confidence intervals.

CC = capsular contracture, FU = follow-up, n = incidences of capsular contracture, N = number of risks.

## 4. Discussion

Vulnerability of the BII to crisis has been previously described, thus termed as the BIC.^[62]^ Moreover, the BIC has been classified into the first BIC (Dow Corning), the second BIC (PIP), and the third BIC (BIA-ALCL).^[63]^ The BII in Korea has also experienced BIC; the first and second BIC in Korea. Of note, however, the BIC in Korea showed a great difference from that in other countries. As described earlier, the manufacturer announced that it would resume the BellaGel business in March of 2023; the safety of finished products of BellaGel/BellaGel SmoothFine has not been further questioned by both the manufacturer and the KMFDS since November 3, 2020.^[46]^ Thus, the manufacturer survived the BIC in Korea and was exempted from the safety issue. Other manufacturers than HansBiomed Co. Ltd. also spontaneously received a letter of indulgence because HansBiomed Co. Ltd. informed the public that even the illegally manufactured breast implants could not be questioned for their safety.

A medical device risk is defined as the likelihood of occurrence of a damage combined with its severity according to the Article II of EU MDR 2017/745. All medical devices are inherently susceptible to a certain level of risks. Therefore, the medical device risk management is a continuous, iterative process in which manufacturers should plan, document and implement risk management strategies that may either eliminate the risk or mitigate the overall severity of it. This is essential for preventing specific harm or risk.^[64]^ Despite the medical device risk management strategies of the manufacturer, however, both a whistleblower and news media played a key role in reporting the first Korean case of a medical device fraud to the public.

It would be worthwhile to analyze the first Korean case of a medical device fraud, namely the second BIC in Korea, based on benchmark cases in risk and crisis management. Tylenol (Johnson & Johnson, New Brunswick) and the Dow Corning SGBI are 2 representative benchmark cases; the former and the latter indicate how to and how not to manage a crisis, respectively. That is, both cases are closely associated with public health and safety issues, although they were managed very differently. As a result, the reputation and image of Dow Coming were considerably impaired.^[65,66]^ Therefore, it remains problematic that the manufacturer of BellaGel/BellaGel SmoothFine continued to lie to the public.^[48]^

To summarize, our results are as follows: first, incidences of CC were 1.79% (3/168), 3.64% (21/577), 8.11% (3/37), and 10.53% (2/19) in the patients receiving Motiva Ergonomix, Sebbin Sublimity, Sebbin Integrity, and Eurosilicone Round Collection, respectively. These differences reached statistical significance (*P* < .05). Second, there were 2 women with rupture after receiving Sebbin Sublimity, although there were no cases of rupture in association with other brands of breast implants. Third, overall CC-free survival was estimated at 681.470 ± 8.314 (95% CI 665.174–697.766) days. By breast implants, it was 708.899 ± 8.595 (95% CI 692.053–725.745), 599.327 ± 6.607 (95% CI 586.378–612.277), 584.941 ± 22.965 (95% CI 539.931–629.952), and 572.492 ± 37.374 (95% CI 499.240–645.745) days in the patients receiving Motiva Ergonomix, Sebbin Sublimity, Sebbin Integrity, and Eurosilicone Round Collection, respectively, in the increasing order. These results indicate that Motiva Ergonomix is a relatively safer device as compared with others in the context of the first Korean case of a medical device fraud, as supported by previous literatures.^[17,22,67,68]^

But our results cannot be generalized because we failed to identify a causal relationship of a risk of CC with differences in the surface topography of between the breast implants. But our results are advocated by a recent study. Doloff et al recently conducted an experimental study to compare differences in immune responses depending on the surface topography of an SGBI between the commercially-available products. These include Motiva Ergonomix Round SilkSurface and VelvetSurface (microtextured; Establishment Labs Holdings Inc.), Mentor Smooth (smooth) and Mentor MemoryGel SILTEX (microtextured) (Mentor Worldwide LLC., Santa Barbara), Microcell (microtextured) and Natrelle INSPIRA Biocell (macrotextured) (Allergan Inc., Irvine). Doloff et al also tested a hypothesis that the human-sized, commercial and miniaturized sample of Motiva Ergonomix Round SilkSurface might alter the kinetics and characteristics of foreign body responses, thus showing that it had long-term effects in inhibiting the fibrosis in New Zealand White (NZW) rabbits (≤1 year) and C57BL/6 mice (≤6 months), respectively. These authors also compared the profile of fibrosis between wild-type and T-cell-deficient C57BL/6 mice, thus showing that there was a significant decrease in the number of macrophages only in wild-type mice. Moreover, Doloff et al also showed that the immune responses of the NZW rabbits and C57BL/6 mice were matched to those seen in human clinical specimens that were collected from the luminal surface of scar capsules formed for 7 months to 11 years in patients receiving an implant-based augmentation mammaplasty.^[69]^ More recent studies have supported the efficacy and safety of Motiva breast implants. Mojo and Nele performed an implant-based augmentation mammaplasty for a consecutive series of a total of 325 patients who were followed up during a mean period of 14 months. These authors reported that Motiva breast implants could provide a favorable choice for balancing both safety and aesthetics in a cohort of patients receiving primary augmentation procedures.^[70]^ Randquist et al performed a total of 1053 primary and secondary breast augmentations, thus conducting a 6-year retrospective follow-up study. These authors demonstrated a low complication rate and safety profile of Motiva breast implants.^[71]^ Lee et al conducted a 3-year single-center retrospective study in a total of 1324 patients comprising 1027 primary cases and 297 secondary ones, thus reporting rates of CC of 1.07% and 4.97%, respectively.^[72]^ The Motiva US IDE is a prospective, single-arm, multicenter, 10-year pivotal study; it collected data from patients receiving augmentation mammaplasty, reconstruction and revisional surgery and then submitted it to the United States Food and Drug Administration. Glicksman et al reviewed the 3-year data about the efficacy and safety of Motiva breast implants. According to these authors, there were low rates of implant-related complications and high rates of patients’ and surgeons’ satisfaction with postoperative outcomes.^[73]^

## 5. Conclusion

According to the National Council for Advanced Manufacturing in the United States, sustainable manufacturing is defined as the manufacturing of sustainable products and the sustainable manufacturing of all the products.^[74]^ The ISO 13485 specifies requirements for a quality management system for organizations required to demonstrate their ability to provide medical devices that consistently meet regulatory requirements, according to which the manufacturer develops and produces under guidelines mandated by the United States Food and Drug Administration.^[75]^

According to Ghadimi and Heavey, the social sustainability of a medical device includes both its safety and a patient’s health.^[76]^ Therefore, the first Korean case of a medical device fraud should be considered as a lack of the social sustainability of the manufacturer of BellaGel/BellaGel SmoothFine despite regulatory requirements enforced by the KMFDS.

In conclusion, our results indicate that Motiva Ergonomix Round SilkSurface is currently a relatively safer device as compared with others in the context of the first Korean case of a medical device fraud. The BII in Korea should be aware of the importance of the social sustainability in manufacturing a device.

## Author contributions

**Conceptualization:** Robert Kim.

**Data curation:** Robert Kim.

**Formal analysis:** Robert Kim.

**Funding acquisition:** Robert Kim.

**Investigation:** Robert Kim.

**Methodology:** Robert Kim.

**Project administration:** Robert Kim.

**Resources:** Robert Kim.

**Software:** Robert Kim.

**Supervision:** Robert Kim.

**Validation:** Robert Kim.

**Visualization:** Robert Kim.

**Writing – original draft:** Robert Kim.

**Writing – review & editing:** Robert Kim.
